# Communicating Health Literacy on Prescription Medications on Social Media: In-depth Interviews With “Patient Influencers”

**DOI:** 10.2196/41867

**Published:** 2023-03-13

**Authors:** Erin Willis, Kate Friedel, Mark Heisten, Melissa Pickett, Amrita Bhowmick

**Affiliations:** 1 University of Colorado Boulder Boulder, CO United States; 2 Health Union, LLC Philadelphia, PA United States; 3 Gillings School of Global Public Health University of North Carolina at Chapel Hill Chapel Hill, NC United States

**Keywords:** social media, social media influencer, pharmaceutical advertising, health literacy

## Abstract

**Background:**

Historically, pharmaceutical companies have struggled with trust and brand reputation among key stakeholders and have adopted innovative marketing strategies to reach patients directly and rebuild those relationships. Social media influencers are a popular strategy to influence younger demographics, including Generation Z and millennials. It is common for social media influencers to work in paid partnerships with brands; this is a multibillion-dollar industry. Long have patients been active in online health communities and social media platforms such as Twitter and Instagram, but in recent years, pharmaceutical marketers have noticed the power of patient persuasion and begun to leverage “patient influencers” in brand campaigns.

**Objective:**

This study aimed to explore how patient influencers communicate health literacy on pharmaceutical medications on social media to their communities of followers.

**Methods:**

A total of 26 in-depth interviews were conducted with patient influencers using a snowball sampling technique. This study is part of a larger project using an interview guide that included a range of topics such as social media practices, logistics of being an influencer, considerations for brand partnerships, and views on the ethical nature of patient influencers. The constructs of the Health Belief Model were used in this study’s data analysis: perceived susceptibility, perceived severity, perceived benefits, perceived barriers, cues to action, and self-efficacy. This study was approved by the institutional review board of the University of Colorado and adhered to ethical standards in interview practice.

**Results:**

As patient influencers are a new phenomenon, it was our goal to identify how health literacy on prescription medications and pharmaceuticals is being communicated on social media. Using the constructs of the Health Belief Model to guide the analysis, 3 themes were identified: understanding disease through experience, staying informed on the science or field, and suggesting that physicians know best.

**Conclusions:**

Patients are actively exchanging health information on social media channels and connecting with other patients who share similar diagnoses. Patient influencers share their knowledge and experience in efforts to help other patients learn about disease self-management and improve their quality of life. Similar to traditional direct-to-consumer advertising, the phenomenon of patient influencers raises ethical questions that need more investigation. In a way, patient influencers are health education agents who may also share prescription medication or pharmaceutical information. They can break down complex health information based on expertise and experience and mitigate the loneliness and isolation that other patients may feel without the support of a community.

## Introduction

### Background

Social media influencers (SMIs) have become ubiquitous on social media and are a growing marketing trend owing to their increasing influence on consumers’ purchasing behaviors [1]. SMIs command audiences across various categories, including travel, fashion, and beauty [2-4], and attract younger generations such as millennials and Generation Z with spending power [5]. Analysts project the influencer marketing industry to reach US $4 billion in 2022, with a US $5.78 return on investment for every US $1 spent on influencer marketing [6]. Once used by brands to raise awareness among audiences in certain demographics, SMIs are now popular tactics included in many organizations’ integrated marketing communication plans.

One such category that has long been thriving but only recently noticed by marketers is that of “patient influencers” [7]. Health Union defines patient influencers as “the most visible and trusted health consumers. They raise awareness, share information and support their communities” [8]. Influencers may also provide companies with a pathway to “leverage the patient experience and expertise in the design, development, and promotion of their products and services.” Patient influencers in different “disease categories” are intimately familiar with the health care system and living with a chronic disease. Many patient influencers can also boast active and engaged followers ranging in size from nano- to microcommunities.

The power of a brand is in consumer perceptions and what they have experienced directly or indirectly about the brand [9]. Although patient influencers may be a new promotional strategy used by pharmaceutical companies, the goal is not quite the same as with lifestyle and pop culture SMIs in product categories such as beauty or fashion. Instead, patient influencers create content and collaborate with pharmaceutical companies to raise awareness and increase health education among peers who share an interest in a particular disease or condition [7]. The content shared by patient influencers focuses on being a patient, having a high quality of life, and sharing experiences to help other patients do the same. Previous research notes that patients often seek out health information, including information on prescription medications and treatment options, from other patients on the web [10,11].

As more consumers turn to social media for news and information [12] and the amount of disinformation and misinformation increases [13,14], it is crucial to consider the health literacy of consumers in the patient influencer relationship. Health literacy is a person’s ability to find and use the information required to make health decisions [15,16]. Similar to previous scholars’ concerns about direct-to-consumer (DTC) pharmaceutical advertising [17-20], patient influencers on social media may communicate complex information that average consumers have difficulty understanding. Although patient influencers are burgeoning in industry practice [21-23], little published research examines this phenomenon. Therefore, this study explored how patient influencers communicate health literacy on pharmaceutical medications on social media to their communities of followers.

### SMI Trend

Today’s digital environment has given rise to a new form of celebrity: SMIs. Unlike traditional celebrities, SMIs establish social media presence as experts in a niche area, sharing content that is personal, authentic, and valuable and growing an audience of followers through consistent content production and synchronous and asynchronous interaction [24-27]. SMIs then use their celebrity capital to collaborate with brands on product messaging and promotion to the influencer’s followers—similar to traditional celebrity endorsements but with more nuanced effort [24].

Specific attributes categorize influencers, including an area of interest; content type; and, most commonly, follower count. Nano-influencers have a small number of followers—typically <1000—but these followers tend to be highly engaged and supportive of the influencer [28]. Microinfluencers range between 1000 and 40,000 followers, most of whom are also highly engaged and often devoted to the influencer [28]. Macroinfluencers, with followers ranging from 40,000 to 1 million, and mega-influencers, with >1 million followers, are often more expensive to work with than other influencer categories [28].

Often, a brand chooses to pursue a partnership with an SMI as a way to leverage the influencer’s audience of followers interested in the influencer’s area of specialized knowledge [25]. Perceived as credible, trustworthy, authentic, and accessible by their followers [24,25] and intentional about engaging with followers in the social media environment [27], SMIs have higher rates of message responsiveness than a brand’s own marketing messages [29]. In addition to content that displays expert knowledge, influencers often express vulnerability by sharing personal content with their followers, who in turn feel emotionally connected to either the message, the influencer, or both [24,26].

### Social Media and Health Behaviors

The Centers for Disease Control and Prevention [30] report that 6 in 10 Americans have at least one chronic disease, and 4 in 10 have 2 or more. Most chronic diseases require patients to practice self-management, which includes taking prescription medications. Patients who seek out medical advice beyond their primary care physician frequently turn to the internet and other patients for answers and social support. A trusted source of web-based health information is online health communities (OHCs). OHCs are a space where patients exchange specialized health and medical information, promote health literacy, interact with a community of shared experience, and gain experience with self-efficacy [31-33]. Simply, self-efficacy is one’s belief in their ability to perform a health behavior [34]. Self-efficacy is the foundation for motivation and accomplishment; unless a person believes that an action will produce the desired outcomes, there is little incentive to act or overcome challenges. Self-efficacy influences the practice of healthy behaviors, cessation of unhealthy ones, and the maintenance of behavior changes regardless of obstacles [35]. Self-efficacy has been used to predict self-management behaviors across chronic diseases [36]. Often, patients seek out OHCs to engage with topics not addressed in their physician’s appointment and learn self-management behaviors from others. Several factors cause patients to seek out health information on the web, including decreasing time spent with a physician, dwindling health literacy among the general public, technology barriers and access issues, lack of adequate health insurance, and the need for social support [31,37-39]. Owing to these inefficiencies, patients often rely on web-based health information to bridge the gaps. This web-based health information–seeking behavior has created a trend of participatory health care that promotes self-efficacy, which allows patients to advocate for themselves and their own treatment experience. In addition, social support is often a contributor to self-efficacy. For example, online communities allow patients with other chronic diseases to share their stories and experiences of their disease. Through the collective sharing of personal experiences, other patients receive more information about their disease, care options, self-management recommendations, and advice from other patients [40,41].

OHCs frequently include opinion leaders, and sometimes those translate to patient influencers on social media platforms. A crucial tenet of influencer content is the perceived connection between SMIs and their followers. Often formed through a combination of vulnerability-laden content and SMI-follower interaction, this connection leads to a perception of similarity between the SMI and their followers, which also leads to higher levels of perceived authenticity [26,42,43]. Scholars refer to “emotional ties” that connect the influencer and followers; this “emotional investment” is key in building online communities [24]. Participating in an online community, especially one centered on health topics and helmed by an influencer perceived as an expert on said topic, can shape followers’ attitudes and behaviors [43]. It can also lead to positive outcomes related to enhanced social support and improved digital health literacy and self-advocacy [31-33].

### Health Belief Model

The Health Belief Model (HBM) was initially devised to explain which beliefs should be addressed by health-promotion efforts aimed at changing negative behaviors among targeted audiences [44]. The model has been used to predict broad health-specific preventative behaviors, self-management actions, and clinic use rates [45-47]. The HBM consists of 6 constructs that offer an evaluative model for measuring messages that motivate behavior or identify potential intervention points in situations where the behavior change initiative is not working effectively. The constructs are *perceived susceptibility*, *perceived severity*, *perceived benefits*, *perceived barriers*, *cues to action*, and *self-efficacy* [48]. The first 4 originated from the initial conception of the HBM; the final 2 were contributions from Social Cognitive Theory [49]. The first is perceived susceptibility, a subjective valuation of the person’s perceived risk. Second, perceived severity estimates a person’s feelings regarding the seriousness of the risk and its effects on medical conditions, such as mortality or prolonged illness, and social relationships, such as familial well-being and access to the community. Third, perceived benefits are a person’s appraisal of the effectiveness of prescribed actions to reduce the risk of disease or death; this construct also considers susceptibility in accepting recommendations. Fourth, perceived barriers factor in obstacles to the adoption of recommended health actions, such as expenses, unexpected hazards, convenience, and time. Fifth, cues to action count the stimuli required to choose and accept the recommended action. Finally, self-efficacy measures an individual’s perceived capabilities and confidence in implementing a discipline of action that supports the proscribed behavior changes [49].

Originally developed in the 1950s, the HBM was conceptualized to explain patient engagement in one-time health behaviors such as cancer screening or immunization [50]. Later, the model was applied to long-term health behavior change such as chronic disease self-management, smoking cessation, and medication adherence [51-53]. The HBM has been used to create effective interventions to change health-related behaviors by targeting specific constructs of the model [54,55]. The last 2 constructs of the HBM were proposed because of the mounting evidence in psychology research on differences in individual decision-making and the influence on health behaviors [48]. Cues to action are necessary for prompting engagement in positive health behaviors and can be internal (eg, physiological cues) or external (eg, media and physicians). Research recognizes that patients’ confidence in their own abilities to effect change in outcomes is critical to health behavior change [48].

Overall, this study applied the constructs of the HBM as patient influencers disseminate content to communities of followers who are actively seeking health information. As younger generations are heavily influenced by social media content [56,57] and more people look to social media and blogs to fill gaps in health care [58], it makes sense that patient influencers have become a credible source of patient information. No research has examined how paid partnerships between patient influencers and pharmaceutical companies are perceived by other users or even what content is being disseminated.

### DTC Pharmaceutical Advertising and Health Literacy

The Food and Drug Administration defined DTC advertising as a pharmaceutical company’s efforts to promote prescription drugs directly to patients [59]. DTC is one of the largest spending categories in US advertising, totaling US $6.58 billion in 2020 [60]. There are arguments for and against the effectiveness of DTC advertising. For example, advertising often places heavy emphasis on emotional appeal without providing more comprehensive health information. In addition, language is often too complex and supersedes the reading skills of the general population [17]. According to recent research, between one-third and one-half of all patients lack the skills to follow instructions related to their medications [61,62]. Health literacy enables patients to find and understand the information so they can then use it to discuss treatment options and care with their physicians [62]. Most adults in the United States have intermediate health literacy skills, whereas more than one-third have basic or below-basic skills [63]. This means that common health behaviors such as reading and understanding prescription medication labels are difficult for many Americans. Higher levels of health literacy allow patients to advocate for themselves as a patient’s health choices are often a direct indication of their health knowledge [64].

Not only is DTC advertising lacking the appropriate context for those who have low health literacy, but it also underrepresents a large portion of the population. In a content analysis of DTC advertisements [17], the dominant ethnicity in most DTC advertisements was White (48%), followed by African American (19.5%), Hispanic (7.3%), Asian American (2.4%), and other (9.8%). An inequitable representation in DTC advertising leads to a lack of self-identity among patients who may be looking to pharmaceutical messaging for validation and information [17]. Often missing too is the understanding of health literacy in a broader context of socioeconomic and cultural differences among patients [65]. Culture plays a role in lifestyle and diet, for example, and in the management of chronic diseases.

Research shows that those with lower health literacy often turn to television, social media, blogs, and celebrities for health information. Scholars conducted a web-based survey (N=600) to assess participants’ health literacy, the sources used to obtain health information, and the level of trust in the health information sources [38]. Participants with lower health literacy were less likely to trust health information from health care professionals but more likely to trust social media and celebrities. Therefore, this study sought to understand the role of patient influencers in communicating health literacy and information on pharmaceutical medications to other patients on social media.

## Methods

Owing to the lack of published literature on patient influencers, this study used in-depth interviews to explore this phenomenon. Scholars argue that in-depth interviews provide much more detailed information than what other data collection methods yield [66].

### Ethics Approval and Informed Consent

This study was approved by and complied with the ethical standards of the University of Colorado Boulder institutional review board for human participant research (21-0472). Informed consent was obtained verbally from each participant before the interview, and participants agreed to the research team’s analysis of the data. Participants were paid US $50 for taking part in the study. Owing to the nature of this study, the interview data were not considered sensitive and, therefore, were kept confidential in a web-based storage folder that was only accessible to the research team. To protect the participants’ confidentiality, names were not used in reporting direct quotes from the interviews.

### Participant Recruitment

The researchers reached out to Health Union to help identify a sample of patient influencers [8]. Health Union is a digital health company that connects patients with health care opportunities. Initially, Health Union provided a curated list of patient influencers to interview. The criteria for participation were being aged ≥18 years, being diagnosed with a disease or health condition, and using social media platforms regularly to discuss health and collaborate with brands. Next, the authors invited patient influencers to participate in the study via email. We then used snowball sampling to recruit more patient influencers to participate in the study. Snowball sampling is when existing study participants refer future participants for research [67].

This study is part of a larger project using an interview guide that included a range of topics such as social media practices, logistics of being an influencer, considerations for brand partnerships, and views on the ethical nature of patient influencers. Refer to Multimedia Appendix 1 for the interview guide. This research focused specifically on the following questions within the interview guide: What brands do you work with? How did those relationships come about? Do people ask you for health information on drug brands, products, or treatments? How do you handle those requests? What are your thoughts on representing pharmaceutical drugs and the power dynamics of this relationship? How do your followers factor into this power dynamic?

Ultimately, the purpose was to understand how patient influencers communicate health literacy related to pharmaceutical medications to their followers. In total, 26 in-depth interviews were conducted via Zoom (Zoom Video Communications) or phone: 18 (69%) were with women, and 8 (31%) were with men; 17 (65%) participants were White, 4 (15%) were Hispanic, 4 (15%) were African American, and 1 (4%) identified as Asian American. The participants had been diagnosed with various conditions, including lupus, fibromyalgia, Parkinson disease, asthma, and HIV. Refer to Table 1 for participant descriptions. A total of 26 interviews was deemed sufficient because of the saturation of data [68]. Interviews were conducted during March and April of the 2022 calendar year. The interviews lasted 72 minutes; the authors analyzed the transcriptions of the recorded interviews.

**Table 1:** Participant descriptions.

Participant ID	Sex	Race or ethnicity	Disease or condition
Participant 1	Female	African American	Diabetes
Participant 2	Female	White	Celiac disease
Participant 3	Female	White	Epilepsy
Participant 4	Female	African American	Lupus
Participant 5	Male	White	Prostate cancer
Participant 6	Female	White	Rare disease
Participant 7	Male	White	Irritable bowel syndrome
Participant 8	Female	White	Fibromyalgia
Participant 9	Male	White	COPD^a^
Participant 10	Male	Hispanic	HIV and anal cancer
Participant 11	Female	White	Multiple sclerosis
Participant 12	Female	White	EDS^b^, MCAS^c^, and POTS^d^
Participant 13	Male	White	FOP^e^
Participant 14	Female	White	Perimenopause, menopause, and postmenopause
Participant 15	Male	Hispanic	Asthma
Participant 16	Female	White	Chronic migraine
Participant 17	Female	White	Chronic migraine
Participant 18	Female	Hispanic	Psoriatic arthritis
Participant 19	Female	Hispanic	Parkinson disease
Participant 20	Female	White	Cystic fibrosis
Participant 21	Male	African American	Diabetes
Participant 22	Female	Asian American	Pulmonary arterial hypertension and congestive heart failure
Participant 23	Female	African American	Multiple myeloma
Participant 24	Female	White	Rare disease
Participant 25	Female	White	Lupus
Participant 26	Male	White	Friedreich ataxia

^a^COPD: chronic obstructive pulmonary disease.

^b^EDS: Ehlers-Danlos syndrome.

^c^MCAS: mast cell activation syndrome.

^d^POTS: postural orthostatic tachycardia syndrome.

^e^FOP: Fibrodysplasia ossificans progressiva.

### Analysis of Data

The analysis was conducted during and after data collection. A researcher conducted all the interviews and made initial notes for analysis. All the researchers then systematically analyzed the transcripts through the data analysis procedures by Miles and Huberman [69]: data reduction, data display, and conclusion drawing. First, the researchers coded interview transcripts from the 3 questions of interest using the constructs of the HBM in data display [48]. Refer to Multimedia Appendix 2 for an example of data display. The constructs were operationalized in terms of information sharing or the information landscape related to a particular disease or condition. Textbox 1 presents the HBM and the final themes of the study.

There were few discrepancies among the researchers in coding the data, and any disagreements were discussed together and a resolution was achieved. Researchers then merged related codes into common themes. This process was done over time through in-depth discussion and reflection on the data and the HBM constructs. To ensure reliability and validity, the research team had regular debriefing sessions to discuss the data [70].

**Health Belief Model constructs and final themes.**

- Perceived susceptibility
  Understanding disease through experience
- Perceived severity
  Understanding disease through experience
- Perceived benefits
  Staying informed on the science or field
- Perceived barriers
  Staying informed on the science or field
- Cues to action
  Suggesting that physicians know best
- Self-efficacy
  Suggesting that physicians know best

## Results

### Overview

This study interviewed patient influencers to understand how they communicate health literacy on pharmaceutical medications on social media to their followers. The HBM guided our data analysis and the development of the findings. The HBM constructs help researchers identify specific factors that influence behavior. As patient influencers are a new phenomenon, it was our goal to identify how health literacy on prescription medications and pharmaceuticals is being communicated on social media. A total of 3 themes were found: *understanding disease through experience*, *staying informed on the science or field*, and *suggesting that physicians know best*. Each theme is discussed in detail in the following sections.

### Understanding Disease Through Experience

All the participants interviewed (26/26, 100%) had been living with their condition or disease for several years and thought of themselves as “expert patients” and “well-versed in the healthcare system.” A few participants (3/26, 12%) could be considered “pioneers” in the OHC and patient influencer space, having been active both on the web and offline in their disease communities for decades. Some members (10/26, 38%) were actively involved with nonprofit organizations related to their condition or disease, organized face-to-face and web-based support groups, and raised money for research. Other patient influencers (3/26, 12%) were active in OHCs long before the advent of social media.

The interviewed participants wanted to help others access information that “wasn’t available when I was diagnosed.” Many welcomed the engagement from their communities, encouraging followers to ask questions or share their own experiences. A total of 23% (6/26) of the participants mentioned using hashtags on Instagram and TikTok so others might find and locate their content. Most patient influencers (24/26, 92%) shared content so others would not be susceptible to a lack of information or education. Participants reported being contacted by other patients very frequently, some of whom were searching for answers and others who had been recently diagnosed or were struggling to find the right treatment. In several cases, racial minority group participants wanted culturally appropriate information that was relevant and helpful to “people like me”:

I spent a lot of time looking for diabetes information that related to me—an African American woman from the South. How to eat. How to cook. How to manage diabetes. And, I didn’t find much. So in 2012, I created the website blackdiabeticinfo.com because I wanted to see an African American that had diabetes that was smiling...I did not see what I needed, so I created it.

Participant 1

Another participant discussed the lack of resources in her region and felt that online patient communities bridged that gap, allowing her to listen to the medical advice others received from physicians and what others in different regions and countries are learning about the disease:

No one knows everything so social media bridges that gap to where you get to learn, you know, learn a lot more stuff. Learn and meet new patients and what their experiences are, because you know, like with SLE...You can have ten patients with the same thing, but it affects each person differently. You are able to speak to survivors in L.A. or New York or out of the country. It allows you to kind of hear, Okay, what are their doctors recommending, and how is it affecting them? What are some things that they try?...I think it bridges the gap and it kind of uncovers the blind spots that we might have had before social media was such a big thing.

Participant 4

In total, 58% (15/26) of the patient influencers acknowledged that the disease experience differs for everybody, so they felt compelled to share their journey on the web. Documenting what it is like to have a chronic disease or rare health condition serves multiple functions for these patient influencers. Repeatedly, the patient influencers interviewed mentioned wanting to raise awareness and share information about their disease or condition to normalize disability and create conversations about such issues:

The reason I started talking about HIV in person and then took it to social media when I had the chance was because there’s still not a lot of talk about Latinos and HIV in the community. Back in 2000, when I was diagnosed and I started volunteering, for every one pamphlet that was in Spanish, there were 20 in English. And so we were so underrepresented, and there was not enough information. And when there was information, it was not culturally appropriate...Latinos, we don’t talk about HIV and AIDS. Latinos, we don’t talk about anal cancer or anything having to do with the butt. And especially when men have to go get checked, screened—they’re not going to do it...It’s important for me to get the word out.

Participant 10

That’s the whole point is to educate. It’s like I want to be giving facts about things, but at the same time, I just want to tell what real life looks like in it. I think there’s just so much stigma around migraines specifically.

Participant 16

A total of 19% (5/26) of the influencers reflected on the loneliness following diagnosis and did not want others to experience that feeling:

There was nobody providing virtual support...So many people are excluded from seeking support because they have a geographical disadvantage, a physical disadvantage, or they may even have a social disadvantage and not want to be in a room with a lot of people.

Participant 5

A few patient influencers (4/26, 15%) had “a method to the madness” related to what content was shared and operated more strategically than others. However, most patient influencers interviewed (22/26, 85%) posted on a whim—when something was happening in life, they thought that others would be interested.

In addition, participants shared their disease journey to motivate others to strive for a better quality of life. Often, patient influencers share content related to disease self-management behaviors, for example, exercise tips, insurance barriers with prescription medications, diet, and nutrition information:

We are the CEO and stakeholder of our health. And, despite all the barriers and obstacles we might come across, we still have control to navigate our health—whether we are 100% healthy or not. We do have some control in that our overall health status and how we approach it really can affect the outcomes of our life...We already have a negative experience with our health if we’re chronically ill. So we have a choice where we can compound it and make it worse with our choices or mindset, or try to make it a little bit better by making better choices and improving our mindset.

Participant 8

As patient influencers share day-to-day content from their lived disease experience, followers from the community can engage—not only with the patient influencer but also with other patients from the disease community. As a result, patients can see the similarities and differences in their disease state and quality of life:

The treatment I went through was very difficult. And within six months of being on that treatment, I realized I needed to advocate. Because it didn’t need to be as difficult as it was if the medical community was tuned in to what was happening.

Participant 5

My advocacy began in the hospital. I wrote my first blog there. It was my way of letting my friends know what happened. I did not have the emotional bandwidth to tell everyone what happened. From here, they were like if this could happen to you...who works out...the tofu-eating one, this could happen to any of us!

Participant 1

Patients have immediate access to others and can receive instant information or engagement. Some patient influencers (5/26, 19%) referred to their followers as “family” or “friends” and felt a sense of responsibility to help and encourage others. As the patient influencers had been active on various platforms for many years, archived content is available to followers, new and old.

### Staying Informed on the Science or Field

Patient influencers reported staying up-to-date on research and pharmaceutical medication development. However, whether the patient influencer shared information about pharmaceuticals and in what way was unique to each participant interviewed. For example, 15% (4/26) of the participants said that they shared news releases from pharmaceutical companies with their followers if the information was relevant to the community. Other patient influencers (3/26, 12%) read medical studies and shared the results in laypeople’s terms on their social media platforms. Sharing this type of information was not prompted by sponsorships or payments from a brand or organization. Instead, the patient influencers wanted to be credible to their followers, which meant staying informed on the latest science in the field:

I feel like I have a unique skill set where I’m not trained in the neuroscience of migraine, but I can read peer-reviewed research and get the gist of it, minus the really technical sort-of-science-y parts. So, I can read it, and when it’s a clinical trial, I understand the treatment control. I understand how to kind of break it down and explain to people what the limitations of different types of research are. I don’t feel like even doctors do that very well for us and certainly not the researchers who are trying to share their information. And so, I guess that’s sort of why I do what I do and what I try to do.

Participant 17

Owing to the awareness and familiarity of many patient influencers within their respective disease communities, it was essential to their credibility and reputation to share only valuable information with their followers:

I think [followers] would like to get authentic and genuine information. Information that isn’t biased. It’s not promotional. I think that’s a big thing that I look at other people’s posts and I can really piece out something that is not very genuine and not authentic. So I think it’s really important to try and maintain authenticity. People really first have to believe in what they’re sharing and do the same, right? So if they’re promoting something, they should be using it and believe it works. It’s not just for the money.

Participant 20

Although many (18/26, 69%) reported working with for-profit brands and pharmaceutical companies, patient influencers felt protective of their communities; they wanted only to benefit followers and support them throughout their disease journey. Therefore, patient influencers were careful never to overstep and “leave my patient lane” (Participant 2).

In total, 19% (5/26) of the patient influencers interviewed did not share any information on pharmaceutical medications. However, several of the participants interviewed (4/26, 15%) had strong opinions about patients sharing pharmaceutical information:

I think I’ve sort of set an unofficial tone or rule that I don’t affiliate with any pharmaceutical companies. I know there are other migraine advocates, influencers who will partner with pharmaceutical companies, and I don’t do it on the advertising end, but I have said I will work with them on the research end if they want to involve me in, if they are doing a survey if they want advising on clinical trial design, if they want to...That part, fine. I will not push their medications.

Participant 17

A total of 15% (4/26) of the patient influencers did not take any prescription medications or had moved off of pharmaceuticals to more natural remedies or regimens. Still other patient influencers (19/26, 73%) would only discuss medications they had experience with, whereas a few (3/26, 12%) would discuss any prescription medication with their community to assist in decision-making. In addition, many influencers (14/26, 54%) referenced the “love-hate” relationship that patients sometimes have with pharmaceutical companies, whereas others (6/26, 23%) applauded the patient-centeredness of some pharmacy brands.

The patient influencers interviewed who took prescription medications (20/26, 77%) often shared their experiences (if asked) with their community of followers. For many patients with chronic diseases, prescription medications are part of managing the disease symptoms. Therefore, much of the information shared on pharmaceuticals is related to symptom management (eg, pain and “flares”) and daily living (or quality of life) or side effects experienced as a result of using the drug:

I tell them my experience. My whole thing is experience...My response is, if your physician thinks you should try it, try it. Here is how it affected me...I don’t push people on anything. But, I do make people aware of the side effects.

Participant 1

Several patient influencers (6/26, 23%) said that in-depth conversations about prescription medications often took place in direct messages. This way, the conversation was kept private, away from the larger community.

Some of the patient influencers (8/26, 31%) discussed obstacles that other patients often encountered and wanted to discuss, including communication with their physician, finding the right specialist, prescription medications, and self-management behaviors:

There’s a lot of information on how to cope. There’s a lot of information about illness and treatment. It’s very difficult to cope every day.

Participant 3

All the patient influencers interviewed (26/26, 100%) were empathetic and expressed compassion for other patients in their community. As the patient influencers felt like “expert patients,” they wanted to mentor and advocate for others:

I hope that by being an advocate, I’m able to influence people to take charge of their health, their bodies.

Participant 10

Everyone just needs to know that they’re not alone and that they’re not crazy in this.

Participant 16

Patient influencers can validate the physical and emotional feelings other patients are experiencing related to their disease. For many with chronic diseases, finding the proper treatment is often challenging, and sometimes, physicians do not have the answers:

I think people come to Twitter when you’ve gotten dismissed by your doctors, or your doctor’s kind of run out of ideas. There’s a big need for better headache and migraine care.

Participant 17

The patient influencers interviewed were willing to help others find the information, resources, and answers they were looking for, which is missing from the health care system:

I [will] post about my treatments. I post a lot about the funny things that happen at doctor’s appointments and things that have happened from diagnosis to now, pre-diagnosis. I [will] talk about medical gaslighting. I talk a lot about bad doctors because I’ve had a lot of those.

Participant 22

I feel like I’m really empathetic in that I can understand how this diagnosis really rocks your world and changes everything. And then you don’t know where to start. I try to really be reassuring and also provide resources, science resources. I’ll provide them links and I’ll provide them [with] studies. And I do all this because I know that it can be really a life-changing diagnosis and be scary.

Participant 2

Patient influencers believe in their mission to advocate for and educate other patients on disease self-management.

### Suggesting That Physicians Know Best

Despite having different approaches to discussing pharmaceutical medications, the patient influencers interviewed all deferred to physicians, telling followers that they should “talk to their doctor.” Depending on the disease or condition, various plausible treatments exist in different disease categories, so the patient influencers’ knowledge of pharmaceutical medications differs significantly. Unanimously, all patient influencers (26/26, 100%) agreed that they would not give medical advice but instead prompt the patient to contact their physician with questions about prescription medications:

It’s more like, Don’t give other people medical advice. You can share what’s worked for you, but no one here is a health professional so you can’t say, “Oh, you shouldn’t be taking 25 milligrams of that, you should be taking 50.” Talk to your doctor. We have rules about things when it comes to talking about treatment stuff.

Participant 17

Depending on what is needed, the patient influencer is able to offer some kind of cue to action. Maybe that is a directive such as “talk to your doctor,” but it could also be a nudge of encouragement or a suggestion for practicing self-management behaviors. All the patient influencers (26/26, 100%) acknowledged that illness and disease present differently for every patient and that it was outside their expertise to offer medical guidance. They also noted that self-management behaviors such as diet and exercise helped immensely with their quality of life:

...I have to reiterate, “I am not a doctor, I’m not giving you medical advice. This is my experience,” and that’s it. I’m giving you my experience with this, this is my opinion...I always start with, “You should talk to a doctor, but this is what I think.”

Participant 22

I won’t diagnose anybody. I won’t say, “That’s IBS” or “That’s not IBS.” I will say, “You definitely have symptoms related to IBS. Have you spoken to your physician about this?” I think it’s really important that people are diagnosed correctly because so many different conditions have overlapping symptoms.

Participant 7

In total, 19% (5/26) of the influencers did not share any prescription drug information simply because they thought it was inappropriate and borderline unethical. Others would only share their experiences with medications they had been prescribed and had taken:

This is my honest recommendation, but you know, not to make any decisions based on my information. Talk to the primary care doctors. I’m not giving them, you know, the treatment plan or medical advice. Just my experience.

Participant 19

Although none of the patient influencers reported negative experiences with followers and their community, they were all conscientious about promoting medications. In addition, several (5/26, 19%) referenced the strict government regulations regarding pharmaceutical advertising.

A total of 12% (3/26) of the patient influencers interviewed pursued further training in advocacy; they noted that patient advocates “are not allowed to give medical information” and should guide followers to seek counsel from their physicians. All the patient influencers interviewed (26/26, 100%) wanted to empower other patients to “live better” and “be actively involved in their own health”:

It’s more valuable information to make better healthcare decisions for themselves and just be more empowered in their own care. It’s overall a general improvement and understand of how healthcare works and how they can take ownership of their health and make more informed decisions together with their doctors.

Participant 20

In total, 62% (16/26) of the patient influencers mentioned during the interview that they ultimately wanted to empower others to be better patients, ask better questions, and make better choices to improve their daily lives. The patient influencers understood that disease is complex and many factors contribute to patients’ well-being:

I hope that people will keep me in mind. Not just the content—although that’s important—but also the quality and the voice I used for them. I hope that they feel less alone and less afraid and more inspired to go out and live the best quality of life they can within their abilities.

Participant 11

Our overall health is going to determine a portion of our lives when you have a chronic condition; it’s always going to be there. But there are other aspects of health or things that I can do to manage and kind of have more days of functioning. The other half is looking at our mental well-being because if you have a negative mindset—that does impact health.

Participant 8

The patient influencers interviewed wanted to motivate action in other patients and facilitate positivity.

The patient influencers mentioned that, as they had so much experience with illness and disease, they could “try and see what does and does not work.” They then felt confident in sharing these experiences with other patients to help them in their disease journey:

Really, my goal is to bring empowerment to patients so they can utilize these tools and resources to improve their own health in some way.

Participant 20

A few patient influencers (4/26, 15%) understood the burden of responsibility from being so public with their diseases and took great care in serving as “expert patients” and community leaders. All the patient influencers interviewed (26/26, 100%) talked about the need for honesty and transparency in their role and how authenticity made them more credible to their followers and the broader disease community:

I really just want to make sure that my audience knows that it’s just what I’m doing because I would hate to give bad advice or anything like that...I don’t want to be telling anyone to do anything and kind of let them have their own choices because that responsibility is big. I don’t want anyone to have an allergic reaction or anything to happen, so I just really want to make sure that everyone is making their own decisions.

Participant 16

When information addressed pharmaceutical medications, the patient influencers were consistent in their responses to followers: “What worked for me may not work for you.” Patient influencers again referred community members to their physicians as experts on prescription drugs.

## Discussion

### Principal Findings

In this study, we interviewed patient influencers to understand how they communicate health literacy on prescription medications to their followers. With many Americans living with chronic diseases [30], it is important to consider the peer-to-peer health information exchanged on self-management behaviors, including pharmaceutical medications. We interviewed 26 patient influencers across “disease categories” who had varying levels of experience with pharmaceutical medications. Using the constructs of the HBM to guide the analysis, 3 themes were identified: *understanding disease through experience*, *staying informed on the science or field*, and *suggesting that physicians know best*.

The patient influencers used social media to share content related to their health—“me just getting through my day.” All the influencers (26/26, 100%) were motivated by their own experiences after diagnosis, not having had enough accessible and culturally appropriate information. One of the inclusion criteria for this study was that the patient influencers collaborate with brands. All the participants (26/26, 100%) collaborated with brands, but not all of those relationships were paid or with pharmaceutical companies. More than half (18/26, 69%) of the influencers interviewed collaborated with a pharmaceutical company in some way: serving on advisory boards, speaking to physicians and researchers, or communicating with key audiences. Although the way patient influencers communicated about pharmaceutical medications differed, as did how they responded to questions about specific drugs, it was agreed among the sample that drug information should only be shared if the patient influencer had direct experience with the medication. When patient influencers communicated about a particular medication, it was usually related to side effects they experienced or “how it worked for me.” Many of the patient influencers (20/26, 77%) mentioned using hashtags as a way to “help others find me” and their specialized content. The remaining influencers (8/26, 31%) collaborated with nonprofit and advocacy organizations or worked with health and medical brands (eg, diabetic socks).

Patient influencers spoke to other patients as experts in disease management after having lived with their condition for such a long time, many experiencing hospitalizations, joint replacements, and insurance challenges, among other things. As health care constraints motivate patients to turn to the internet and social media for health information, platforms such as Instagram and TikTok climb in popularity [57]. Often, patients seek other patients’ experiences and knowledge as an additional avenue for social support and validation. Several of the participants (4/26, 15%) talked about “medical gaslighting” and the negative experiences they had had with the health care system. As a participant said, “Doctors don’t know everything.” Researchers point out that brands leverage influencers’ reputations and specialized knowledge to influence purchase behaviors in target audiences [25]. Similarly, pharmaceutical companies collaborate with patient influencers for their specialized knowledge and influence on their community of followers.

In total, 77% (20/26) of the patient influencers who did share their experiences with pharmaceutical medications with their community of followers agreed that transparency was key. They did not want to work with a pharmaceutical company if it would restrict their messaging. The patient influencers wanted to be an accurate, trustworthy source for their followers and did not ever want to mislead other patients. The patient influencers understood that everybody—and every medication—is different. Many patient influencers interviewed (15/26, 58%) researched disease topics, new pharmaceutical medications, and research trials. Patient influencers wanted to share this type of health information so that other patients could become educated and make positive health decisions. Followers often develop an emotional connection through the influencers’ shared content [24,26]. The emotional connection between the patient influencers and followers has not yet been studied. However, it stands to reason that the emotional connection might be stronger than the typical follower-influencer relationship, especially in nano- and microinfluencer communities where followers receive higher social support from those with the same chronic disease.

This project applied the HBM to the data analysis to understand how patient influencers communicate health literacy on prescription medications. The 6 constructs in the HBM are perceived susceptibility, perceived severity, perceived benefits, perceived barriers, cues to action, and self-efficacy [48]. As all the patient influencers interviewed (26/26, 100%) discussed the lack of information available after their diagnosis, they did not want others to be susceptible. The lack of information had transformed the patient influencers’ lives and affected their treatment decision-making. The patient influencers create and share the type of content they “could have used a long time ago. I want other patients to have that information.” They often sought to minimize the loneliness they initially felt regarding their health care. Social media channels allow patients to self-compare with patient influencers and “see and hear what I’m doing.” Some patient influencers (10/26, 38%) shared their experiences—positive and negative—with pharmaceutical medications, often occurring out of sight via direct messages that are not visible to the whole community. The patient influencers shared their challenges and perceived barriers with their community of followers. Discussing common, shared, or potential barriers to treatments and disease management helps other patients navigate their health care journeys and provide a place for social support. The patient influencers frequently discussed wanting to help other patients overcome their challenges and “to live a good life with this disease.” There were several cues to action communicated by the patient influencers, including prompting other patients to learn about treatment options and talk to their physician for prescription drug information. Over and over in the interviews, we heard the following from patient influencers: “What works for me may not for you.” These patient influencers were adamant about not giving medical advice, nor did they want their followers to misconstrue experience for advice. A few of the patient influencers interviewed in this study (3/26, 12%) mentioned completing patient advocacy training and knew not to overstep government regulations. All the patient influencers (26/26, 100%) wanted to help their followers and raise awareness about the disease by providing a safe and supportive community. Despite enduring many health challenges, these patient influencers expressed self-efficacy to engage in self-management and “take care of my health.” By sharing their success, patient influencers may increase their followers’ self-efficacy to practice self-management, including medication adherence.

Patient influencers are domain experts and share content with followers; however, they are not consistently compensated for their efforts. Often, pharmaceutical companies call upon patient influencers to share their personal experiences with advisors and other stakeholders. These patient influencers share their disease experience for the benefit of others. As many patient influencers are also active in OHCs, they have developed reputations in their “disease category”; other patients view them as knowledgeable. Their specialized content makes their communities of followers often close-knit and very active.

Similar to traditional DTC advertising [71,72], the phenomenon of patient influencers raises ethical questions that need more investigation. In a way, patient influencers are interactive health education agents who may also share prescription medication information. They can break down complex health information based on expertise and experience and mitigate the loneliness and isolation that other patients may feel without the support of a community. In addition, patient influencers are able to meet other patients on their disease journey as they frequently have shared experience. Patient influencers discuss perceived benefits and barriers according to their own knowledge and experience, offering their experiences with disease symptoms and medication side effects. Although patients can compare and contrast, physicians are the gatekeepers of prescription medications and other medical treatments; therefore, it is important for patients to advocate for themselves and understand their disease and treatment options. Patient influencers help facilitate self-efficacy for other patients to talk to their physicians and seek a high quality of life.

The effects of the practice of patient influencers are not yet understood. As this is a new and rapidly growing phenomenon, little research has examined patient influencers in the academic literature. Consumer perceptions and what they have experienced directly or indirectly about a brand influence behavior [9]. SMIs have not slowed in popularity and continue to be lucrative marketing investments [6]. Pharmaceutical companies have quickly adopted social media strategies to target and connect directly with patients, shape consumers’ brand perceptions, and build relationships with younger audiences in niche market segments. The current status of health literacy in the United States is concerning, especially coupled with the digital literacy of younger generations [16]. However, patient influencers potentially bridge a gap in the health care system and offer information and advice in laypeople’s terms or in a way that is culturally relevant; frequently, there is an exchange and opportunity to ask questions when patient influencers connect with other patients on the web without fear of stigma or shame. Many chronic disease self-management education programs use peer educators to deliver the curriculum or program [36], and so, perhaps patient influencers provide opportunities to reimagine patient education and health education programs.

### Limitations

This study is not without limitations. We conducted in-depth interviews, and qualitative data cannot be generalized. Our participants were ethnically diverse and were diagnosed with a range of chronic diseases and conditions. No generalizations based on ethnicity or specific disease can be drawn from the data. The snowball sampling method is also limited in that participants are from the same network.

### Future Research

Future research needs to define “patient influencers” and their particular characteristics and attributes compared with what we typically think of as SMIs. More work needs to focus on diverse patient populations or specific disease categories. Future research should seek to test the effectiveness of patient influencers in improving self-management behaviors and their influence on patients’ treatment decisions. Research should investigate how pharmaceutical companies determine which patients to work with and the nature of compensation. Finally, further work should examine the regulations related to social media marketing of prescription drugs and the monitoring of content.

### Practical Implications

There are several theoretical and practical implications of this research. With regard to the HBM, this study adds to our understanding of peer-to-peer communication regarding pharmaceutical medications. Although patients are exchanging “lived” experiences of specific medications, including side effects and disease symptoms, there is potential for patient influencers to engage in health education and promotion. As data analytics processes improve, there may be opportunities to use web-based patient data to better predict self-management behaviors. There are also several practical implications. Nonprofit and health organizations may collaborate with patient influencers to disseminate targeted health information so as to improve health literacy in specific patient groups. Health education and promotion might be designed to take advantage of the digital and social media platforms available, including the patient communities actively using these channels. Finally, more government attention needs to be paid to this new phenomenon of patient influencers and how this practice is being implemented.

### Conclusions

Patient influencers may be a viable option to address issues regarding diversity and health disparities and clinical trial representation and access. Patients have been on the web exchanging health information for decades. Patient influencers and OHCs have also been around and active for decades; it is just that, in recent times and culture, health care is becoming more patient-centric, and patients are becoming more engaged in the decision-making process. Government regulations must be updated in the face of new platforms and spaces such as direct messages where patients are meeting and sharing health information or best practices, and industry guidelines (at the very least) should be created to protect all stakeholders. In addition, regulators should scrutinize transparency and disclosure practices concerning digital platforms and unique functionalities such as long-form video, disappearing content, and direct messaging.

## Appendix

Multimedia Appendix 1 Interview guide.

Multimedia Appendix 2 Coding schema in data display.

## Abbreviations

DTC: direct-to-consumer
HBM: Health Belief Model
OHC: online health community
SMI: social media influencer

## Data Availability

Interview transcripts are available upon request from the lead researcher.
