# Supplementary material for: Communicating Health Literacy on Prescription Medications on Social Media: In-depth Interviews With “Patient Influencers”
Source: J Med Internet Res. 2023 Mar 13;25:e41867. doi: 10.2196/41867 (PMC10131845; doi:10.2196/41867)
Supplement: Multimedia Appendix 1 [file jmir_v25i1e41867_app1.docx]

**Interview Instrument - Script**

Hello! Thank you for being here today. Is it okay if I record this interview?

This is an in-depth interview that explores the ethics and morality of patient influencers. I sent you an informed consent document, did you have a chance to read it?

Great! Did you understand the document?

Do you have any questions about the study and the interview today?

Do you agree to participate in this study?

If you want to stop the interview at any time, you can do so without penalty. Just let me know.

Let’s get started with some of my questions.

1. What draws you to the topic of health? e.g., why have you chosen to be on social in the space?
2. Do you consider yourself an “influencer”? What does that entail for you? What expectations do others have of you?
3. Do you like introducing new brands and products/treatments to your followers?
4. Did you start your account with the intention of being a patient advocate or influencer? If so, why?
   1. *Possible probe*: Would you say that you are a patient advocate? If yes, can you describe what being a patient advocate means to you?
   2. *Possible probe*: Do you like helping people by providing them with information on many kinds of products?
5. How would you describe the health needs of your followers? Prompt with health-related language if necessary.
6. Why is social media a good platform to respond to these needs? What are the limitations of social media?

You know this because you do it, but social media is a full-time job. Let’s talk about the logistics for a few minutes.

1. Do you usually create the content you share or is content provided to you? How do you decide what content to share?
   1. *Possible probe*: What are some of the most common decisions you make in your job as an influencer? Does this include choosing what topic to post on? Does this include what images to post or what captions to write?
2. Are there certain things or certain topics of posts that are your favorite to post? Why?
3. Are there certain things or certain topics of posts that you prefer not to post? Why?
4. What brands do you work with? How did those relationships come about? What health partners do you work with
   1. *Possible probe*: Do you think you were sought out based on your demographics? Age? Gender? Race/ethnicity? Part of the country you’re from?
5. Have you ever declined to partner with a health brand or organization? Why? Or Why not?

Now, moving into your decision-making and how you decide on content, let me ask you a few questions in that regard.

1. In working with partners do you ever consider the cost of the product or the activity associated with the partner? Why? Or Why not?
2. How do you feel about having to make these decisions? Which of these decisions are ethical in nature? Can you explain?
3. Do you rely on anyone when making these decisions? Who? How and why? How do your followers factor into these decisions? What’s the nature of your relationship with followers?
4. What choices do your followers make because of your content? What do your followers do with the information you post? Are these good choices? Can you explain? What are the most common reactions or comments to your post?

Now, you are different than what’s typically known as an influencer because you are also a patient. I’d like us to explore that a little bit now.

1. What role does empathy and compassion play in your work as a patient influencer? Can you give an example?
2. Have you been thanked for what you have posted? Or has someone has been angry with what you have posted? Why? And how did you handled it? Have you ever severed ties or had to block followers and why?
3. Do people ask you for information on drug brands, products or treatments? How do you handle those requests?
4. Do any of your posts target specific types of people? (women, moms, younger people, ethnic background, etc.?). Do you check their analytics? What are the most important information that you get from their analytics? How do your partners “evaluate” their relationships? Are they supposed to achieve a certain number of engagements? Followers?
5. What are your thoughts on representing pharmaceutical drugs and the power dynamics of this relationship? How do your followers factor into this power dynamic?
6. How would you characterize your relationship with your followers? Do you consider your followers friends?
