# Supplementary material for: Communicating Health Literacy on Prescription Medications on Social Media: In-depth Interviews With “Patient Influencers”
Source: J Med Internet Res. 2023 Mar 13;25:e41867. doi: 10.2196/41867 (PMC10131845; doi:10.2196/41867)
Supplement: Multimedia Appendix 2 [file jmir_v25i1e41867_app2.docx]

**Multimedia Appendix 2.** Coding schema in data display.

| HBM^a^ construct | Participant quote | Theme |
| --- | --- | --- |
| Perceived susceptibility | “*I spent a lot of time looking for diabetes information that related to me*^b^—an African American woman from the South: how to eat, how to cook, how to manage diabetes, and *I didn’t find much*. So in 2012, I created the website blackdiabeticinfo.com because I wanted to see an African American that had diabetes that was smiling...I did not see what I needed, so I created it.” [Participant 1] | Understanding disease through experience |
| Perceived susceptibility | “*My advocacy began in the hospital*. I wrote my first blog there. It was my way of letting my friends know what happened. I did not have the emotional bandwidth to tell everyone what happened. From here, they were like if this could happen to you...who works out...the tofu-eating one, *this could happen to any of us*!” [Participant 1] | Understanding disease through experience |
| Perceived severity | “No one knows everything so social media bridges that gap to where you get to learn, you know, learn a lot more stuff. *Learn and meet new patients and what their experiences are, because you know, like with SLE, you can have 10 patients with the same thing but it affects each person differently*. You are able to speak to survivors in L.A. or New York or out of the country. It allows you to kind of hear, okay, what are their doctors recommending, and how is it affecting them? What are some things that they try? I think it bridges the gap and it kind of uncovers the blind spots that we might have had before social media was such a big thing.” [Participant 4] | Understanding disease through experience |
| Perceived severity | “*There was nobody providing virtual support*...So many people are excluded from seeking support because they have a geographical disadvantage, a physical disadvantage, or they may even have a social disadvantage and not want to be in a room with a lot of people.” [Participant 5] | Understanding disease through experience |
| Perceived severity | “*The treatment I went through was very difficult*. And within six months of being on that treatment, I realized I needed to advocate. Because it didn’t need to be as difficult as it was if the medical community was tuned in to what was happening.” [Participant 5] | Understanding disease through experience |
| Perceived severity | “We are the CEO and stakeholder of our health. And, despite all the barriers and obstacles we might come across, *we still have control to navigate our health*—whether we are 100% healthy or not. We do have some control in that our overall health status and *how we approach it really can affect the outcomes of our life*...We already have a negative experience with our health if we’re chronically ill. So we have a choice where we can compound it and make it worse with our choices or mindset, or try to make it a little bit better by *making better choices* and improving our mindset.” [Participant 8] | Understanding disease through experience |
| Perceived susceptibility | “The reason I started talking about HIV in person and then took it to social media when I had the chance was because *there’s still not a lot of talk about Latinos and HIV in the community*. *Back in 2000* when I was diagnosed and I started volunteering, for every one pamphlet that was in Spanish, there were 20 in English. And so we were so underrepresented, and there was not enough information. And when there was information, *it was not culturally appropriate*...Latinos, we don’t talk about HIV and AIDS. Latinos, we don’t talk about anal cancer or anything having to do with the butt. And especially when men have to go get checked, screened—they’re not going to do it...It’s important for me to get the word out.” [Participant 10] | Understanding disease through experience |
| Perceived susceptibility | “That’s the whole point is to *educate*. It’s like I want to be giving facts about things, but at the same time, *I just want to tell what real life looks like in it*. I think there’s just so much stigma around migraines specifically.” [Participant 16] | Understanding disease through experience |
| Perceived barrier | “I feel like I have a unique skill set where I’m not trained in the neuroscience of migraine, but I can read peer-reviewed research and get the gist of it, minus the really technical sort-of-science-y parts. So, I can read it, and when it’s a clinical trial, I understand the treatment control. *I understand how to kind of break it down and explain to people what the limitations of different types of research are. I don’t feel like even doctors do that very well for us and certainly not the researchers who are trying to share their information*. And so, I guess that’s sort of why I do what I do and what I try to do.” [Participant 17] | Staying informed on the science |
| Perceived benefit | “I think [followers] would like to get *authentic and genuine information. Information that isn’t biased*. It’s not promotional. I think that’s a big thing that I look at other people’s posts and I can really piece out something that is not very genuine and not authentic. So I think *it’s really important to try and maintain authenticity*. People really first have to believe in what they’re sharing and do the same, right? So if they’re promoting something, they should be using it and believe it works. It’s not just for the money.” [Participant 20] | Staying informed on the science |
| Perceived benefit | “I think I’ve sort of set an *unofficial tone or rule that I don’t affiliate with any pharmaceutical companies*. I know there are other migraine advocates, influencers who will partner with pharmaceutical companies, and I don’t do it on the advertising end, but I have said *I will work with them on the research end if they want to involve me in, if they are doing a survey if they want advising on clinical trial design, if they want to*...That part, fine. I will not push their medications.” [Participant 17] | Staying informed on the science |
| Perceived barrier | “I tell them my experience. *My whole thing is experience*...My response is, if your physician thinks you should try it, try it. Here is how it affected me...I don’t push people on anything. But, I do *make people aware of the side effects*.” [Participant 1] | Staying informed on the science |
| Perceived barrier | “*There’s a lot of information on how to cope. There’s a lot of information about illness and treatment. It’s very difficult to cope every day*.” [Participant 3] | Staying informed on the science |
| Perceived benefit | “I hope that by being an advocate, *I’m able to influence people to take charge of their health, their bodies*.” [Participant 10] | Staying informed on the science |
| Perceived benefit | “*Everyone just needs to know that they’re not alone* and that they’re not crazy in this.” [Participant 16] | Staying informed on the science |
| Perceived barrier | “*I think people come to Twitter when you’ve gotten dismissed by your doctors, or your doctor’s kind of run out of ideas*. There’s a big need for better headache and migraine care.” [Participant 17] | Staying informed on the science |
| Perceived barrier | “*I [will] post about my treatments. I post a lot about the funny things that happen at doctor’s appointments and things that have happened from diagnosis to now, pre-diagnosis. I [will] talk about medical gaslighting. I talk a lot about bad doctors because I’ve had a lot of those*.” [Participant 22] | Staying informed on the science |
| Perceived benefit | “I feel like *I’m really empathetic* in that I can understand how this diagnosis really rocks your world and changes everything. And then you don’t know where to start. *I try to really be reassuring and also provide resources, science resources.* I’ll provide them links and I’ll provide them [with] studies. And I do all this because *I know that it can be really a life-changing diagnosis and be scary*.” [Participant 2] | Staying informed on the science |
| Cues to action | “It’s more like, Don’t give other people medical advice. You can share what’s worked for you, but no one here is a health professional so you can’t say, ‘Oh, you shouldn’t be taking 25 milligrams of that, you should be taking 50.’ Talk to your doctor. We have rules about things when it comes to talking about treatment stuff.” [Participant 17] | Suggesting that physicians know best |
| Cues to action | “...I have to reiterate, ‘I am not a doctor, I’m not giving you medical advice. This is my experience,’ and that’s it. I’m giving you my experience with this, this is my opinion...I always start with, ‘You should talk to a doctor, but this is what I think.’” [Participant 22] | Suggesting that physicians know best |
| Cues to action | “I won’t diagnose anybody. I won’t say, ‘That’s IBS’ or ‘That’s not IBS.’ I will say, ‘*You definitely have symptoms related to IBS. Have you spoken to your physician about this?*’ I think it’s really important that people are diagnosed correctly because so many different conditions have overlapping symptoms.” [Participant 7] | Suggesting that physicians know best |
| Cues to action | “This is my honest recommendation, but you know, not to make any decisions based on my information. *Talk to the primary care doctors. I’m not giving them, you know, the treatment plan or medical advice. Just my experience.*” [Participant 19] | Suggesting that physicians know best |
| Self-efficacy | “It’s more valuable information to make better healthcare decisions for themselves and just *be more empowered in their own care. It’s overall a general improvement and understand of how healthcare works and how they can take ownership of their health* and make more informed decisions together with their doctors.” [Participant 20] | Suggesting that physicians know best |
| Self-efficacy | “I hope that people will keep me in mind. Not just the content—although that’s important—but also the quality and the voice I used for them. *I hope that they feel less alone and less afraid and more inspired to go out and live the best quality of life they can within their abilities.*” [Participant 11] | Suggesting that physicians know best |
| Self-efficacy | “Our overall health is going to determine a portion of our lives when you have a chronic condition; it’s always going to be there. *But there are other aspects of health or things that I can do to manage and kind of have more days of functioning. The other half is looking at our mental well-being because if you have a negative mindset—that does impact health*.” [Participant 8] | Suggesting that physicians know best |
| Self-efficacy | “Really, *my goal is to bring empowerment to patients so they can utilize these tools and resources to improve their own health in some way.*” [Participant 20] | Suggesting that physicians know best |
| Cues to action | “I really just want to make sure that my audience knows that it’s just what I’m doing because I would hate to give bad advice or anything like that...I don’t want to be telling anyone to do anything and kind of let them have their own choices because that responsibility is big. I don’t want anyone to have an allergic reaction or anything to happen, so *I just really want to make sure that everyone is making their own decisions.*” [Participant 16] | Suggesting that physicians know best |

^a^HBM: Health Belief Model.
